# Assessing the Permeability of Engineered Capillary Networks in a 3D Culture

**DOI:** 10.1371/journal.pone.0022086

**Published:** 2011-07-07

**Authors:** Stephanie J. Grainger, Andrew J. Putnam

**Affiliations:** Department of Biomedical Engineering, University of Michigan, Ann Arbor, Michigan, United States of America; The University of Akron, United States of America

## Abstract

Many pathologies are characterized by poor blood vessel growth and reduced nutrient delivery to the surrounding tissue, introducing a need for tissue engineered blood vessels. Our lab has developed a 3D co-culture method to grow interconnected networks of pericyte-invested capillaries, which can anastamose with host vasculature following implantation to restore blood flow to ischemic tissues. However, if the engineered vessels contain endothelial cells (ECs) that are misaligned or contain wide junctional gaps, they may function improperly and behave more like the pathologic vessels that nourish tumors. The purpose of this study was to test the resistance to permeability of these networks *in vitro*, grown with different stromal cell types, as a metric of vessel functionality. A fluorescent dextran tracer was used to visualize transport across the endothelium and the pixel intensity was quantified using a customized MATLAB algorithm. In fibroblast-EC co-cultures, the dextran tracer easily penetrated through the vessel wall and permeability was high through the first 5 days of culture, indicative of vessel immaturity. Beyond day 5, dextran accumulated at the periphery of the vessel, with very little transported across the endothelium. Quantitatively, permeability dropped from initial levels of 61% to 39% after 7 days, and to 7% after 2 weeks. When ECs were co-cultured with bone marrow-derived mesenchymal stem cells (MSCs) or adipose-derived stem cells (AdSCs), much tighter control of permeability was achieved. Relative to the EC-fibroblast co-cultures, permeabilities were reduced 41% for the EC-MSC co-cultures and 50% for the EC-AdSC co-cultures after 3 days of culture. By day 14, these permeabilities decreased by 68% and 77% over the EC-fibroblast cultures. Co-cultures containing stem cells exhibit elevated VE-cadherin levels and more prominent EC-EC junctional complexes when compared to cultures containing fibroblasts. These data suggest the stromal cell identity influences the functionality and physiologic relevance of engineered capillary networks.

## Introduction

Success within the tissue engineering field continues to be limited due to the inability to form a functional vasculature capable of supplying oxygen and nutrients to sustain tissue growth and metabolism [1], [2], [3]. Fabrication of constructs larger than 200 µm in thickness has been mostly unsuccessful, with large, hollow organs or avascular tissues being two exceptions [4]. Tissues thicker than this threshold are unable to overcome the limits of diffusion to properly nourish the tissue. Several possible solutions to overcome this hurdle have been proposed and explored in the literature over the past decade. One promising option involves the delivery of combinations of pro-angiogenic factors with precise spatial and temporal resolution in order to recruit host vasculature [5]. However, this approach can be limited by the fact that the half-lives of these factors are often very short, thereby limiting their bioactivity, and by the fact that even multiple combinations of factors cannot fully recapitulate the complex milieu of pro-angiogenic factors presented to cells *in vivo*. An alternative approach is to provide the cell types that are required for new vessel formation, so that they can directly deliver the appropriate cocktail of required pro-angiogenic cues as necessary. Amongst the most recent possible solutions to overcome this hurdle is the idea of creating prevascularized tissues, which contain networks of vessels formed *in vitro* that can self-organize and anastamose with the host vasculature *in vivo* shortly after implantation [6], [7].

Efforts to create prevascularized tissue constructs typically involve co-cultures of ECs and a supporting mesenchymal cell type, which is intended to act as a pericyte coat, within a 3D biomaterial scaffold [8], [9]. Several cell types have been shown to promote capillary morphogenesis and adopt periendothelial locations, including fibroblasts [10], SMCs [11], MSCs [12], and AdSCs [13], [14]. While all of these cell types appear to promote capillary formation, it is unclear if each type yields capillaries whose functional properties are similar to those of healthy, mature capillaries. Previous work from our group shows that different stromal cells promote capillary sprouting in fibrin hydrogels via different proteolytic enzymes [15], [16], [17], but the functional consequences (if any) of this difference are unknown.

**Figure 1:**
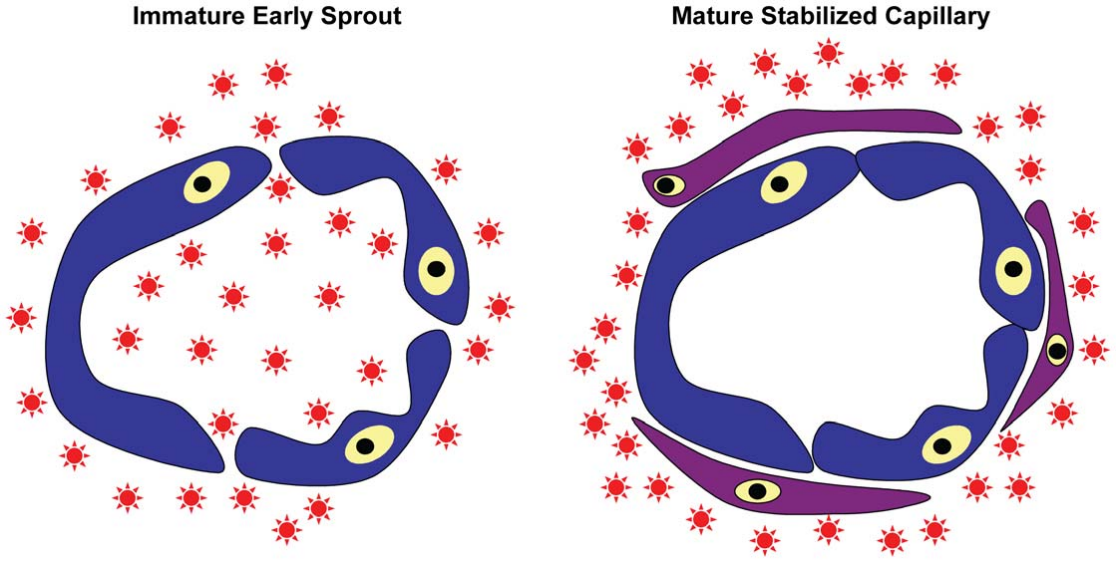
Model of inverse permeability used to determine capillary functionality kinetics in a 3D culture. Texas Red-conjugated dextran (70 kDa) is added to the bulk gel for 30 minutes and allowed to freely diffuse. Early capillary sprouts, which lack mature cell-cell junctions between the endothelial cells that form the tubules, will easily allow the tracer to be transported from the tissue space into the interior of the capillary. By contrast, mature capillaries that are stabilized by pericytes and possess more mature cell-cell junctions are capable of excluding the tracer from the lumen.

This study focuses on the functional differences of capillary networks assembled from ECs and these varying mesenchymal cell types as stabilizing pericytes, using permeability as one marker of vessel functionality. A model of inverse permeability was developed, where a function-defining tracer was added to the bulk tissue and allowed to diffuse throughout. The premise behind this model is that immature capillaries with incomplete cell-cell junctions will be unable to regulate permeability, thus allowing tracer to enter the capillary lumens [18], [19], [20], [21], [22]. If the capillaries present are mature, competent cell-cell junctions between endothelial cells, as well as the presence of stabilizing pericytes, will block tracer from entering the hollow lumens of the capillaries (Figure 1).

## Methods

### Ethics Statement

Human umbilical vein endothelial cells (HUVECs) were harvested from fresh umbilical cords following a previously established protocol [8]. The cords were obtained via a process considered exempt by the University of Michigan's institutional review board because the tissue is normally discarded, and no identifying information is provided to the researchers who receive the cords.

### Cell Culture

HUVECs were grown in fully supplemented Endothelial Growth Medium (EGM-2, Lonza, Walkersville, MD). Normal human lung fibroblasts (NHLFs, Lonza) were cultured in Media 199 (Invitrogen, Carlsbad, CA) supplemented with 10% FBS, 1% penicillin/streptomycin (Mediatech, Manassas, VA), and 0.5% gentamicin (Invitrogen) at 37°C and 5% CO_2_. Mesenchymal stem cells (Lonza) and adipose-derived stem cells (Invitrogen) were cultured in Dulbecco's modified Eagle Medium (DMEM, Sigma-Aldrich, St. Louis, MO) supplemented with 10% FBS, 1% penicillin/streptomycin (Mediatech), 0.5% gentamicin (Invitrogen) at 37°C and 5% CO_2_. NHLFs, MSCs, and AdSCs were all used prior to passage ten. Cells were cultured in monolayers until reaching 80% confluency and serially passaged using 0.05% trypsin-EDTA treatment.

### Tissue Construct Assembly

Four million HUVECs (p3) were harvested and coated on 10,000 presterilized Cytodex (Sigma-Aldrich, St. Louis, MO) microcarrier beads (131–220 µm diameter) in 5 ml of EGM-2 in an inverted T-25 flask over a 4 hour incubation period with mild agitation every 30 minutes. After 4 hours, 5 ml of fresh EGM-2 were added and the total volume was transferred to a fresh T-25 flask for incubation in standard cell culture position overnight. A 2.5 mg/ml bovine fibrinogen (Sigma-Aldrich) solution was made in serum-free EGM-2 and filtered through a 0.22 µm syringe filter. A 500 µl solution of the fibrinogen with 5% FBS was mixed with 50 pre-coated beads and polymerized by addition of 10 µl of thrombin (50 U/ml, Sigma- Aldrich) in a single well of a 24-well tissue culture plate. The mixture was incubated at 25°C for 5 minutes, and then at 100% humidity, 37°C and 5% CO_2_ for 25 minutes. 25,000 NHLFs were plated on top of each gel after polymerization, and 1 ml of fresh EGM-2 was then added to the top of the gel. Media was changed every other day. Identical constructs were also made using AdSCs and MSCs. For some applications, stromal cells were distributed throughout the gel constructs rather than in a monolayer on top of the constructs.

### Tissue Construct Assembly for Confocal Microscopy

The standard protocol was followed as described above, with the following alterations. Beads were coated and fibrinogen solution was made in the standard manner. A 125 µl solution of the fibrinogen with 5% FBS and 10,000 NHLFs were mixed with 25 pre-coated beads and polymerized by addition of 2.5 µl of thrombin (50 U/ml, Sigma Aldrich) in a single well of a #1 chambered 8-well coverglass. The mixture was incubated at 25°C for 5 minutes, and then at 100% humidity, 37°C and 5% CO_2_ for 25 minutes. 350 µl of fresh EGM-2 were then added to the top of the gel and media was changed every day. Identical constructs were also made using AdSCs, and MSCs.

### Permeability Assay

Selectively permeable mature capillaries are known to be impermeable to dextrans over a molecular weight of 65 kDa, so a Texas Red-conjugated dextran (λ_ex/em_ of 595/615 nm) with a molecular weight of 70 kDa was chosen (Invitrogen) [23]. This dextran molecule also contains free lysines, and is therefore fixable in formalin. All samples were directly incubated at 25°C for 30 minutes with a 5% dextran solution in phosphate buffered saline (PBS), and then excess dextran was removed by 3 washes of 10 minutes each with PBS. In some experiments, histamine (Sigma-Aldrich) was added to capillary cultures prior to dextran addition and culture termination via fixation at a concentration of 100 µM and allowed to incubate for 5 minutes [22]. Assays were continued as previously described above by addition of dextran, fixation, staining, and 3D confocal imaging.

### Staining

After the constructs were allowed to incubate for a specified time period (3, 5, 7, 10, 12, or 14 days), samples were fixed in 10% formalin (Sigma Aldrich) at 4°C for 20 minutes. Formalin was removed with three 5 minute washes in PBS. Non-specific protein binding was eliminated with a 1 hour block in antibody diluting solution (0.1% Triton X-100 in TBS (TBS-T), with 2% bovine serum albumin) (AbDil), and then washing was repeated 3 times with TBS-T. Human CD-31 primary antibody (Dako, Carpinteria, CA) was diluted 1∶50 in AbDil and incubated at 4°C overnight. After incubation, excess antibody was removed with 3 washes in TBS-T of 20 minutes each, and then a final overnight wash. Secondary antibody (AlexaFluor 488 goat anti-mouse, Invitrogen) at a 1∶400 dilution in Abdil was incubated for 2 hours. Excess antibody was removed with 3 washes in TBS-T of 20 minutes each and then a final overnight wash at 4°C. Cells were also stained with DAPI nuclear stain (Invitrogen) at a 1∶10,000 dilution in PBS for 10 minutes and washed for 10 minutes.

For VE-cadherin staining, a 5 minute permeabilization step with 0.25% Triton X- 100 in TBS-T was included prior to blocking. Human VE-cadherin primary antibody (BV9 clone, Santa Cruz Biotechnology, Santa Cruz, CA) was diluted 1∶50 in AbDil and incubated at 4°C overnight. All subsequent steps were as described above for hCD31 staining, with the exception of a secondary antibody dilution of 1∶100.

### Western Blotting

After the constructs were allowed to incubate for a specified time period (3, 5, 7, 10, 12, or 14 days), samples were lysed using RIPA lysis buffer (50 mM Tris-HCl pH 7.6, 150 mM NaCl, 1% Triton X-100, 0.5% sodium deoxycholate, 0.1% SDS), homogenized, and two cycles of 30 second sonication and 20 second vortexing were completed. Lysates were cleared via centrifugation at 14,000 x g for 10 minutes at 4°C. Total protein concentration was determined via bicinchoninic acid assay (Thermo Fisher Scientific, Waltham, MA). Samples were boiled, then equal amounts of total protein were loaded for all co-culture conditions into a 10% tris-glycine gel (Invitrogen) and then electrophoretically separated for 3.5 hours. Proteins were then transferred onto a poly(vinylidine fluoride) membrane and probed via mouse anti-human VE-cadherin antibody (Santa Cruz Biotechonology). After washing, secondary horseradish peroxidase-conjugated anti-mouse antibody was incubated on the membrane (Santa Cruz Biotechnology). Protein expression was then visualized via an enhanced chemiluminescence detection system.

### Quantification of Total Network Length & Number of Vessel Segments

Prior to bead coating, HUVECs were labeled with cell tracker dye, SP-DiIC_18_(3) (Invitrogen) and tissues were constructed as described above. Low magnification fluorescent images (4x) were taken at days 3, 7, 10, and 14 by tracking the same beads throughout growth via MetaMorph (Molecular Devices, Sunnyvale, CA). Images were then quantified via the Angiogenesis Tube Formation module of MetaMorph to track the total network length and the total number of vessel segments per bead. Upper and lower limits were defined to exclude beads and nodes from the quantification. Five individual beads were imaged per cell type for each of three separate experiments for a total of fifteen images per experimental condition.

### Confocal Microscopy

All samples were imaged using an Olympus IX81 spinning disk confocal microscope equipped with a 100-W high pressure mercury lamp (Olympus, Center Valley, PA) and a Hammamatsu camera (Bridgewater, NJ) along a vertical z-stack of the entire diameter of each capillary with 0.5 µm optical slices to visualize the location of dextran within the interior/exterior of each capillary. Single confocal images were taken for proof of concept dextran localization on a Zeiss LSM 510-Meta laser scanning confocal microscope.

### Design of an image analysis program for quantification

**Figure 2:**
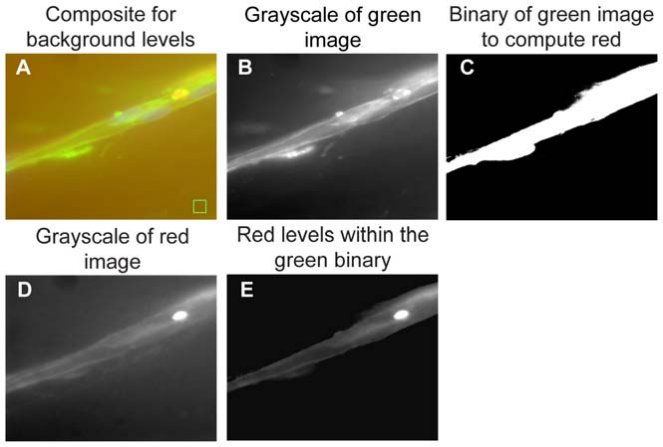
Pixel comparison algorithm. In Panel A, the algorithm makes a 3D overlay of the images of each channel. The user defines the location of a background box. In Panel B, each green image from the 3D z-stack is depicted in gray scale. In Panel C, the maximum projection of the green z-stack is converted to a binary image after the background (defined in Panel A) is eliminated. In Panel D, the portion of the red 3D z-stack that corresponds to the same area depicted in the green image is shown in gray scale as a maximum projection. In Panel E, only those pixels from the red image in Panel D that overlap with the green pixels in Panel C are shown. The pixel intensities from the image in Panel E are tallied and output as a ratio of red:green.

A customized MATLAB algorithm was designed to quantify the amount of dextran that was able to penetrate into the capillaries. All acquired confocal z-stacks were overlayed and compiled into a 3D overlay of the images of each channel (Texas Red-dextran, AlexaFluor 488-hCD31, DAPI). A sample image can be found in Figure 2, Panel A. A 100-by-100 pixel box was placed in this image to read the background level of green. Each green image from the 3D z-stack is then seen in gray scale (Panel B). The maximum projection of these green images from the z-stack is converted to a binary image. The cutoff for this binary image is the background level which was previously defined in Panel A. The portion of the red 3D z-stack that lies within the green image is seen in Panel D as a gray scale maximum projection image. In Panel E, only pixels from the red image in Panel D that overlap with the image in Panel C are shown. The pixel intensities from the image in Panel E are then tallied and output as a ratio of red:green.

### Statistical Analysis

All statistical analyses were performed using GraphPad Prism (GraphPad Software, La Jolla, California). Data are reported as mean ± standard deviation. One way analysis of variance (ANOVA) was performed with a Newman-Keuls multiple comparison post-hoc test. Statistical significance was assumed when p<0.05.

## Results

### Assay Development and Validation

During angiogenesis, new capillary sprouts form following initial budding from a source vessel, and become more elongated, branched, and stabilized by supporting pericytes over time. An important functional hallmark of new capillaries is the reformation of basement membrane and the ability to regulate transport across the vessel wall. In this study, a 3D cell culture model of angiogenesis was combined with a quantitative algorithm to develop a metric of capillary permeability and maturation. At specific time points, fluorescent dextran was added to the culture medium and allowed to incubate for a short period of time, after which the cultures were fixed. The localization of the dextran on the inside or outside of the capillary also provided an indirect determination of the timing of pericyte-EC associations (Figure 1). To better define the edges of capillaries for our quantitative algorithm (Figure 2), HUVECs that sprouted from the microcarrier beads were also stained with hCD31 antibodies.

**Figure 3:**
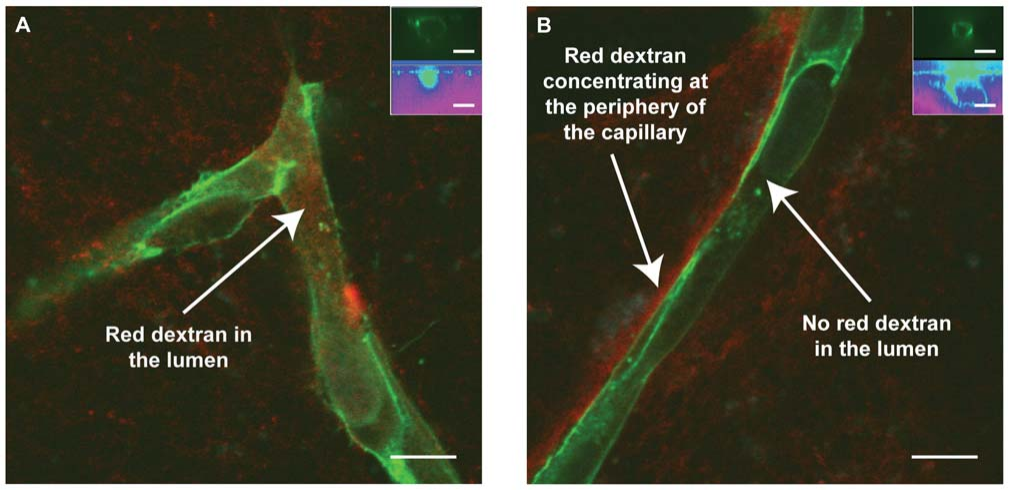
Dextran tracer localization via confocal microscopy. 60x confocal microscopy images of days 5 (A) and 10 (B) capillaries after 70 kDa dextran tracer addition (red), fixation with 10% formalin, and human CD31 staining (green) of HUVECs. Inset images: HUVECs (top) and Dextran (bottom) show hollow lumens with or without dextran. A) Red tracer is present within the lumens of the capillaries, demonstrating a low resistance to permeability. B) Red tracer is excluded from the lumens of the capillaries, demonstrating an increased resistance to permeability as the capillaries mature over time.

Within the first few days after the 3D cell culture was initiated, HUVECs coated on the microcarrier beads began to sprout. At relatively early time points (day 5), dextran tracer added to the culture medium permeated throughout the entire 3D gel, and could even be found within the newly-formed lumens of the nascent vessels (Figure 3A), consistent with the idea that these vessels are particularly immature and lack the structural features necessary to regulate permeability. As the sprouts matured, dextran transport across the vessel wall was limited as shown by the reduced amount of fluorescent dextran that diffused from the bulk tissue into the lumens of the capillaries. As the cultures matured further (day 10), the dextran was nearly completely excluded from the lumens of the capillaries (Figure 3B), especially as seen in cross-section (Figure 3B, inset).

**Figure 4:**
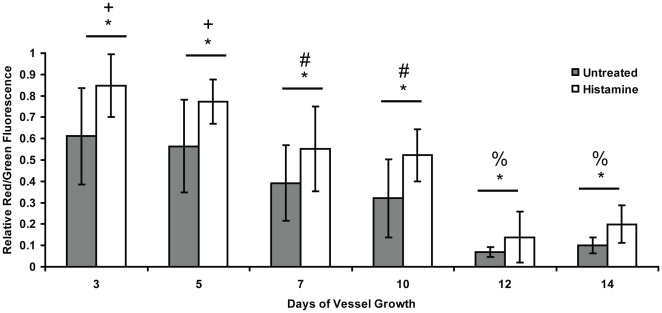
Assay validation using histamine to modulate capillary permeability. Histamine treatment quantitatively modulates permeability of the engineered capillary networks, as expected. In this graph, the * symbol indicates statistical significance (p<0.05) between the black and white bars (−/+ histamine) at each time point. The +, #, and % symbols indicate statistically significant differences (p<0.005) between the data marked by these symbols at certain time points and all other time points marked with different symbols. For example, the values at day 3 and day 5 (for both −/+ histamine) are significantly different from the values at all of the other time points, but not from each other.

To validate this model system and our quantitative algorithm, cultures were treated with histamine, a known modulator of capillary permeability [22]. The output of the quantitative algorithm was a ratio of red (dextran) to green (CD31) fluorescence for each capillary. After treatment with histamine, cultures showed a marked increase in permeability across all time points (Figure 4). At day 3, histamine triggered a 39% increase in permeability over untreated controls, even though the vessels were relatively immature in terms of their structure. By day 14, when the vessels had fully matured, tracer accumulation within the capillaries following histamine treatment was nearly twice that of untreated controls, indicating that the nascent capillaries are indeed capable of regulating their permeability in response to a physiologic stimulus, and that our algorithm is capable of detecting such changes.

### Control of Permeability Evolves as Capillaries Mature

**Figure 5:**
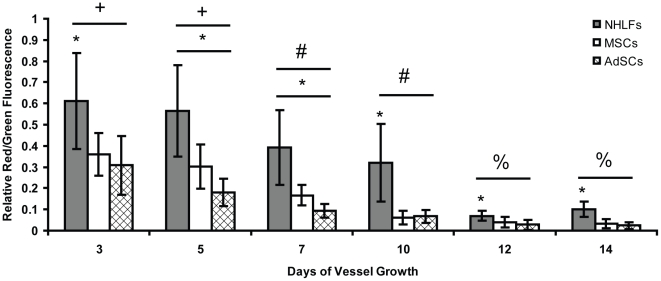
Quantification of permeability using a customized algorithm. Quantification generated via the customized MATLAB algorithm to determine the amount of Texas Red-conjugated dextran within the capillary interior at each time point. An isolated * symbol indicates statistical significance (p<0.05) between data points (NHLF vs. MSC vs. AdSC) at any one particular time point, whereas a bar beneath the * symbol indicates statistical significance (p<0.05) across all 3 experimental groups at each time point. The +, #, and % symbols indicate statistically significant differences (p<0.005) between the data marked by these symbols at certain time points and all other time points marked with different symbols.

Next, we used this newly validated approach to characterize the kinetics with which the capillaries formed within our 3D cell culture model mature with time. The baseline model system involves the use of stromal fibroblasts (i.e., NHLFs) distributed throughout the 3D gels as the source of stabilizing pericytes. At early time points (days 3 and 5), dextran tracer accumulated within the lumens of capillaries, as shown by the high ratio of red (dextran) to green (the boundary of the capillaries, marked by hCD31 staining) fluorescence (Figure 5, black bars). These high levels of permeability gradually decreased with time as the capillaries matured. At intermediate time points (days 7 and 10), the red:green ratio decreased by up to 48% when fibroblasts were used as the stromal cells (Figure 5, black bars). By later time points (days 12 and 14), almost all of the dextran was being excluded from the capillary lumens (89% decrease from initial levels).

### Alteration of Stromal Cell Type Modulates Capillary Permeability

When stromal cell types other than fibroblasts were used to stimulate capillary morphogenesis in this assay, we observed significant differences in the regulation of vessel permeability. Specifically, MSCs and AdSCs produced stable capillaries much more quickly than did the fibroblasts, as shown by a significant reduction in the ratio of red to green fluorescence (Figure 5, white and crosshatched bars). At day 3, capillaries composed of HUVEC-MSC co-cultures and HUVEC-AdSC co-cultures showed reductions in red to green fluorescence of 50 and 59%, respectively, compared to the values from HUVEC-fibroblast co-cultures. This indicated that far less of the dextran tracer crossed the capillary walls in cultures containing these multipotent stromal cells compared to those containing fibroblasts. At later time points, these capillaries also regulated their permeability much more tightly than their fibroblast counterparts, with highly significant decreases of 68% and 77% in the red:green fluorescence ratios by day 14 for the MSC-mediated capillaries and the AdSC-mediated capillaries, respectively, relative to the fibroblast controls.

**Figure 6:**
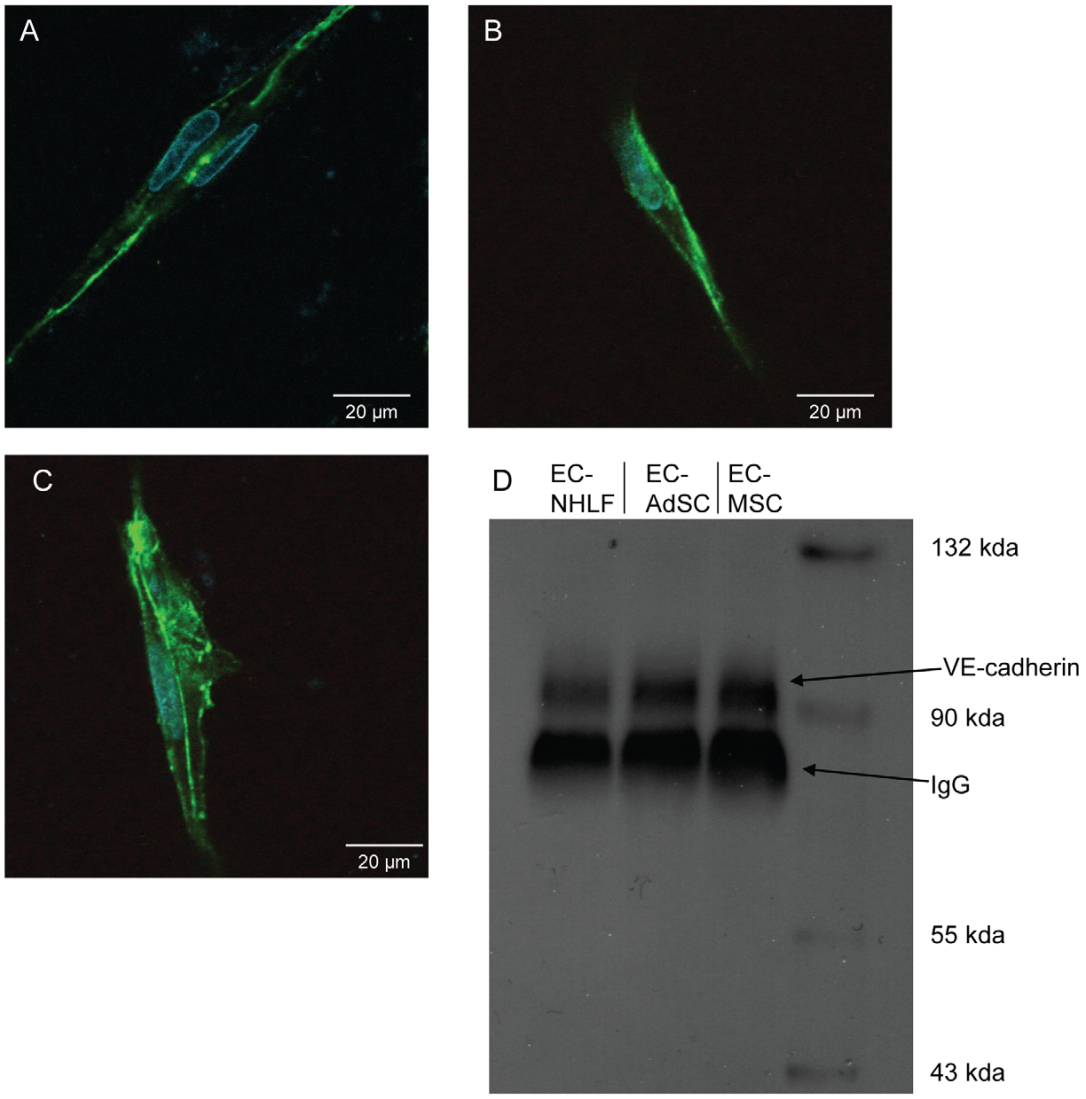
VE-cadherin organization and expression vary as a function of stromal cell type. A–C) 20x confocal microscopy images of day 14 capillaries from co-cultures of EC-NHLF (A), EC-AdSC (B), and EC-MSC (C) after staining for VE-cadherin (green) and DAPI (blue). D) Western blot showing expression of VE-cadherin within each of the co-culture conditions.

To better understand mechanistically how permeability might be differentially controlled by each stromal cell type, the protein levels and organization of VE-cadherin were assessed. Immunofluorescent staining of VE-cadherin revealed that the cell-cell junctions between neighboring endothelial cells of capillary sprouts were more pronounced in EC-AdSC and EC-MSC co-cultures, whereas the EC-NHLF co-cultures showed evidence for more sparse EC-EC junctions (Figure 6, Panels A–C). Cultures containing stem cells also possessed elevated levels of VE-cadherin protein via Western blotting (Figure 6, Panel D).

### Stem Cells Produce Slower Growth Kinetics

**Figure 7:**
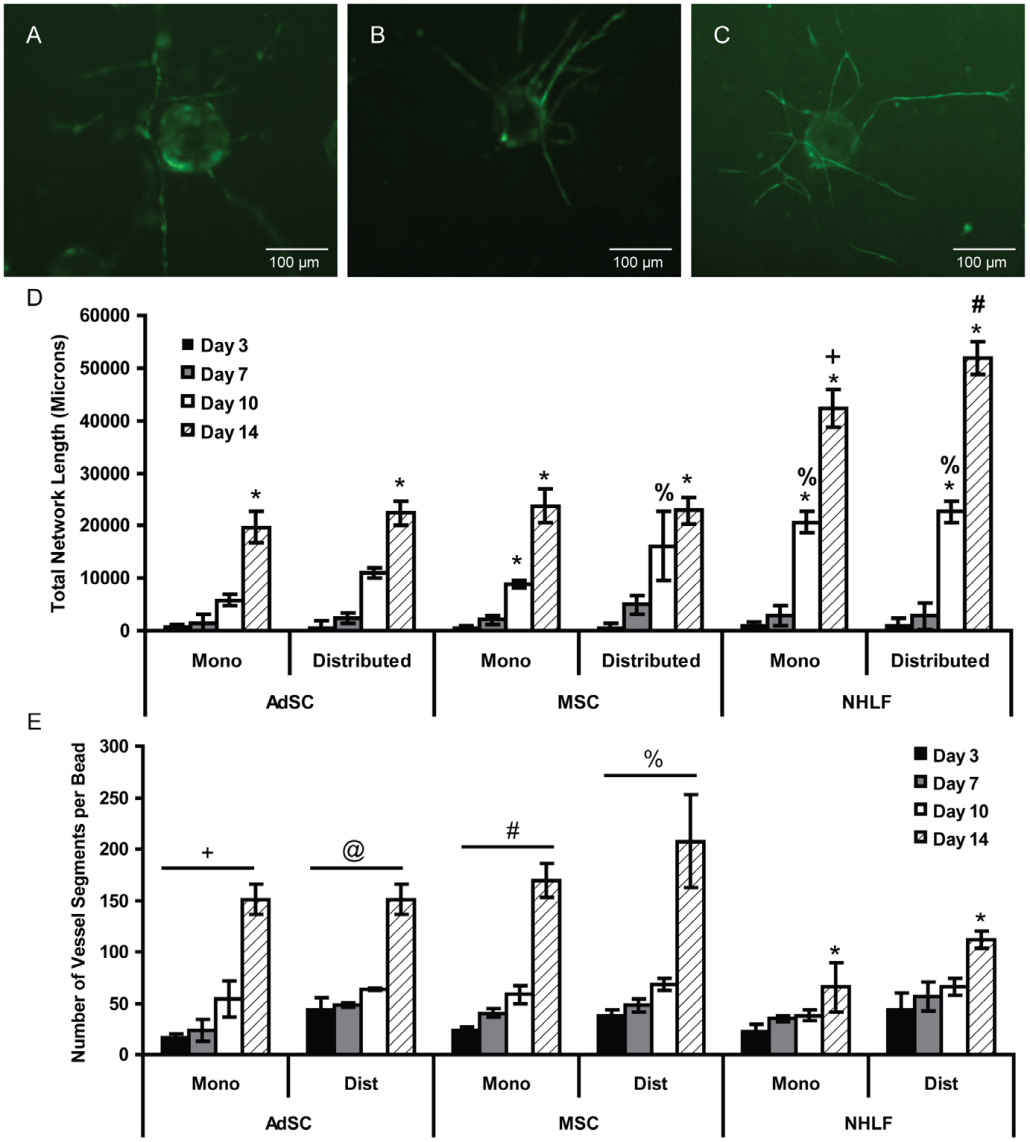
Capillary growth kinetics vary by stromal cell type. A–C) Representative 4x images of fluorescently-tagged HUVECs forming capillary networks in co-culture with A) AdSCs, B) MSCs, C) NHLFs. D) Comparison of growth kinetics for several stromal cell types in the bead assay. “Mono” denotes the stromal cells were cultured as a monolayer on top of the fibrin gel, a fixed distance away from the HUVEC-coated beads; “distributed” indicates the stromal cells were distributed throughout the entire 3D gel. E) Comparison of the number of vessel segments per network for each stromal cell type. The * symbol indicates statistical significance (p<0.05) between time points within a given culture condition. The +, #, %, and @ symbols indicate statistical significance (p<0.005) for a particular culture condition at a given time point versus the other culture conditions. For example, the total lengths of the capillary networks induced by distributed NHLFs are significantly different than those induced by the monolayer NHLFs and those induced by all of the other cell types and distributions (denoted by #).

Prior work has shown that the presence of stromal cells is required for capillary morphogenesis [24]; however, the results presented here indicate that the identity of the stromal cells may also significantly impact the functional properties of the vessel networks. We hypothesized that these observed differences in vessel functionalities may be related to the rates at which the vessel networks form. To further explore this possibility, the kinetics of capillary growth were assessed as a function of stromal cell identity (i.e., NHLF vs. MSC vs. AdSC) and their location relative to the capillaries (i.e., as a monolayer a fixed distance away from the HUVECs vs. distributed throughout the 3D gel) (Figure 7). When cultured as monolayers a fixed distance above the HUVEC-coated beads, AdSCs and MSCs produced capillary networks that were only 47 and 56% of the total lengths of the capillary networks induced by monolayer fibroblast cultures (Figure 7D). When stromal cells were distributed throughout the fibrin gels to more closely approximate physiologic conditions, total network lengths increased for all stromal cell types in comparison to constructs in monolayer cultures; however, a similar trend was seen where cultures with stem cells produced networks with much shorter total lengths than those capillaries cultured with NHLFs as a source of stromal cells. AdSC and MSC capillary cultures produced only 43 and 45% of the total network length of fibroblast cultures after 14 days of growth (p<0.005) (Figure 7D).

To further characterize how the identity of the stromal cells affected capillary network growth, the total number of vessel segments per network was also quantified (Figure 7E). After 14 days, EC-MSC and EC-AdSC co-cultures produced averages of 207 and 150 segments per dextran bead, while EC-NHLF co-cultures produced fewer segments per network, an average of 111. When these values were used in conjunction with total network lengths, EC-NHLF co-cultures had an average segment length of 463 µm after 14 days of culture, whereas EC-MSC and EC-AdSC co-cultures had average segment lengths of only 110 µm and 148 µm, respectively. Together, these data demonstrate that EC-NHLF co-cultures formed longer, more spindly, and less-branched capillary networks than did co-cultures containing either stem cell type.

## Discussion

Capillaries, composite systems of endothelial cells tubules that are sparsely covered with pericytes and a supporting extracellular matrix, serve many functions within the body, most notably acting as selectively permeable barriers which allow nutrients and oxygen into the surrounding tissues, as well as extracting waste products [25], [26]. Larger vessels must also react to vasoactive stimuli, tightening or expanding when required, in order to maintain a level blood pressure and to supply oxygenated blood to all organs and tissues, regardless of the external conditions [27]. In order for a vessel to be classified as functional, it must also differentiate to fit into the proper portion of the hierarchical structure [28]. Arteries and arterioles function as a pipeline to deliver oxygen and nutrients to tissues that are further away, while capillaries provide large surface areas for molecular exchange of gases, nutrients, and wastes within the tissue space [29]. Recreating capillary blood vessels remains a limiting hurdle to the successful clinical implementation of engineered tissues [30].

In the case of implanted tissue constructs, the presence of functional capillary networks has typically been defined by the presence of red blood cells within lumen-containing structures on histological sections; however, observations of leakiness and edema *in vivo* have created a need for a more quantifiable metric of capillary functionality, beyond the mere presence of red blood cells [6], [27], [30], [31]. Furthermore, in diseases such as cancer, phenotypic changes in the pericyte coat and its dissociation from the endothelial cell layer, have been implicated in alterations in vessel permeability [18], [32], [33]. Studies using fluorescent tracers and/or non-invasive imaging methods have enabled vessel perfusion and functionality to be assessed *in vivo* [21]. *In vitro*, established permeability metrics typically measure transendothelial resistance in a Transwell system in which endothelial cells are cultured in a 2D monolayer [34]. However, comparable approaches to assess vessel functionality in 3D are lacking, which in turn creates an inability to determine if a promising approach to either promote or inhibit vessel formation will also alter vessel functionality. Classical assays of resistance across a monolayer cannot be easily applied to a 3D system where the luminal diameters are less than 20 µm; thus a new approach was required.

To address this limitation, here we have described an *in vitro* method for measuring changes in permeability in the capillary vessels created in a previously established 3D tissue culture model [8]. By quantifying the transport of a fluorescent tracer from the tissue space into the vessel lumens, we have effectively shown that permeability can be inversely measured by the use of 3D confocal microscopy and the design of a customized pixel comparison algorithm. The method detected decreases in capillary permeability over time, and showed that some stromal cell types are better able than others to promote the formation of robust, stable networks after 2 weeks of culture. Higher amounts of fluorescent tracer inside the capillary lumens were detected at early time points, which we attribute to a lack of competent cell-cell junctions between endothelial cells and to the fact that pericytes are not yet stabilizing the capillaries. After the constructs are allowed to mature for longer periods of time, and the stromal cells make direct contact with the capillary sprouts as we have previously shown [15], a direct reduction in the amount of tracer was observed in the capillary lumens.

The quantitative algorithm developed here demonstrated the capability to discern quantifiable differences in permeability induced by histamine, a known modulator of permeability, in both immature and mature capillary sprouts. This result is particularly striking given that the lumenal surfaces of neovessels within our system are not perfused via fluid, which has been shown to influence permeability in other systems [35]. We have also shown here that the use of different stromal cell types in our capillary culture system produces capillaries of varying phenotypes and maturation levels as determined both visually and quantifiably using our permeability assay. In recent studies, we have also reported that the mechanisms by which endothelial cells proteolytically degrade the surrounding matrix to form new capillary tubules depends on the identity of the stromal cells in HUVEC-stromal cell co-cultures [15], [17]. Whether or not these mechanistic differences account for the functional differences observed here remains to be seen.

We have typically used fibroblasts as the stromal cell type in our *in vitro* models of angiogenesis with the intent of mimicking the wound healing environment [9]. Fibroblasts are recruited into the wound bed from surrounding host tissues in order to facilitate faster healing and regeneration of the vasculature. Fibroblasts function quickly to promote granulation tissue formation via collagen and fibronectin deposition [36], and their ability to drive capillary growth in our model system is consistent with their known roles in wound healing *in vivo*. However, we observed in this work that fibroblasts promote the formation of vessels characterized by a reduced capacity to control permeability, especially at early time points, relative to those promoted by multipotent stromal cells. However, we observed in this work that fibroblasts promoted the formation of vessels characterized by a reduced capacity to control permeability, especially at early time points, relative to those promoted by multipotent stromal cells. Fibroblasts also promoted vessels at a significantly faster rate than MSCs and AdSCs in our model, but the resulting vessels were longer, less branched, and possessed fewer cell-cell adherens junctions between ECs than those formed from co-cultures containing stem cells. These observations suggest that the multipotent MSCs and AdSCs do in fact modulate their functionalities as pericytes to produce capillaries that more closely mimic the physiology of healthy vasculature, with more controlled permeabilities due perhaps to an increase in EC-EC adherens junctions, while the fibroblasts promote a vasculature more reminiscent of pathologic angiogenesis. Therefore, an especially important conclusion with respect to promoting the neovascularization of engineered tissues has emerged: the choice of which cell type to deliver with endothelial cells to create new vasculature may determine in part whether the capillaries are physiologically healthy or not. Explicit studies to address this question *in vivo* are ongoing.
